# Low *ARID1A* Expression is Associated with Poor Prognosis in Hepatocellular Carcinoma

**DOI:** 10.3390/cells9092002

**Published:** 2020-09-01

**Authors:** Sun Young Yim, Sang Hee Kang, Ji-Hyun Shin, Yun Seong Jeong, Bo Hwa Sohn, Soon Ho Um, Ju-Seog Lee

**Affiliations:** 1 Department of Internal Medicine, Korea University College of Medicine, Seoul 136-701, Korea; eug203@korea.ac.kr (S.Y.Y.); umsh@korea.ac.kr (S.H.U.); 2 Department of Surgery, Korea University College of Medicine, Seoul 136-701, Korea; kasaha1@korea.ac.kr; 3 Department of Systems Biology, The University of Texas MD Anderson Cancer Center, Houston, TX 77030, USA; jishin@mdanderson.org (J.-H.S.); ysjeong@mdanderson.org (Y.S.J.); BSohn@mdanderson.org (B.H.S.)

**Keywords:** *ARID1A*, hepatocellular carcinoma, genomics, prognosis

## Abstract

AT-rich interactive domain 1A (*ARID1A*) is one of the most frequently mutated genes in hepatocellular carcinoma (HCC), but its clinical significance is not clarified. We aimed to evaluate the clinical significance of low *ARID1A* expression in HCC. By analyzing the gene expression data of liver from *Arid1a*-knockout mice, hepatic *Arid1a*-specific gene expression signature was identified (*p* < 0.05 and 0.5-fold difference). From this signature, a prediction model was developed to identify tissues lacking *Arid1a* activity and was applied to gene expression data from three independent cohorts of HCC patients to stratify patients according to *ARID1A* activity. The molecular features associated with loss of *ARID1A* were analyzed using The Cancer Genome Atlas (TCGA) multi-platform data, and Ingenuity Pathway Analysis (IPA) was done to uncover potential signaling pathways associated with *ARID1A* loss. *ARID1A* inactivation was clinically associated with poor prognosis in all three independent cohorts and was consistently related to poor prognosis subtypes of previously reported gene signatures (highly proliferative, hepatic stem cell, silence of Hippo pathway, and high recurrence signatures). Immune activity, indicated by significantly lower IFNG6 and cytolytic activity scores and enrichment of regulatory T-cell composition, was lower in the *ARID1A*-low subtype than *ARID1A*-high subtype. Ingenuity pathway analysis revealed that direct upstream transcription regulators of the *ARID1A* signature were genes associated with cell cycle, including *E2F* group, *CCND1,* and *MYC,* while tumor suppressors such as *TP53, SMAD3,* and *CTNNB1* were significantly inhibited. *ARID1A* plays an important role in immune activity and regulating multiple genes involved in HCC development. Low-*ARID1A* subtype was associated with poor clinical outcome and suggests the possibility of *ARID1A* as a prognostic biomarker in HCC patients.

## 1. Introduction

The prevalence of hepatocellular carcinoma (HCC) worldwide is 750,000 cases per year, making it the seventh most common cancer globally. With a mortality rate similar to the prevalence rate, HCC is the second most common cause of death from any type of cancer [1]. Despite the progress in our understanding of the pathogenesis of HCC and our efforts in screening those at high risk, only one third of patients are candidates for curative or life-extending loco-regional therapies, such as resection, transplantation, or local ablation. For patients whose disease is diagnosed at an advanced stage, curative treatment is not an option [2].

For HCC patients who are not candidates for locoregional therapies, the oral multi-kinase inhibitor sorafenib is currently the only drug approved in the U.S. that has been shown to prolong overall survival. However, most patients develop disease progression after an initial response to sorafenib; the average radiologic progression occurs at 5 months of treatment [3]. Since the release of sorafenib 10 years ago, only one drug, lenvatinib, was proved to be non-inferior to sorafenib as first line treatment [4], while most of the targeted therapies in phase III trials first-line have failed to improve on sorafenib [5,6]. Effective treatment for advanced HCC is a substantial unmet medical need.

One of the important roadblocks to treatment efficacy is the lack of understanding of the mechanism of HCC progression. Improving our understanding of this mechanism would allow the development of therapies directly targeting that mechanism and identification of patients who would respond to those therapies [7]. While the METIV-HCC study enrolled patients based on biomarker analysis, specifically overexpression of MET, most other studies had no biomarker for patient enrollment [8]. Most of the multi-kinase inhibitors targeting BRAF, vascular endothelial growth factor receptor (VEGFR), or platelet-derived growth factor receptor (PDGFR) failed to show improvement in the overall survival for patients with HCC [5,6,9] as they were not able to overcome resistance or tolerance after the use of sorafenib. We propose, therefore, that new drugs that employ alternative mechanisms of action in HCC have the potential to increase the survival rate.

The role of epigenetics in cancer progression has been emphasized ever since the introduction of cancer genome-wide sequencing, which revealed significant alteration in genes responsible for modifying chromatin structure. Chromatin remodeling genes AT-rich interactive domain 1A (*ARID1A*) and BRCA1-associated protein 1 (*BAP1*) are highly mutated in HCC, along with other well-known genes such as *TERT, TP53*, and *CTNNB1* [10]. Accumulating data have implicated *ARID1A* as a key member of the switching defective/sucrose non-fermenting (SWI/SNF) complex, which acts as a tumor suppressor in a broad spectrum of human cancers [11]. However, Sun et al. reported that *ARID1A* has a context-specific role in liver cancer, whereby elevated *ARID1A* promotes tumor initiation, while reduced *ARID1A* in established tumors increases metastasis in mouse models [12].

Because the role of *ARID1A* in the liver is still controversial, with few studies in clinical settings [13,14], we aimed to evaluate the clinical significance of an *ARID1A* gene signature in patients who had already developed HCC. The signature was derived from mouse gene expression data released by Sun et al., which demonstrated that deletion of *Arid1a* in mice that had undergone hepatectomy potentiated greater regeneration capacity than the mice with wild-type (WT) *Arid1a* [15]. Since increased proliferation is one of the steps in hepatocarcinogenesis, we focused on the role of *ARID1A* mutation-related genes in predicting the outcome of HCC. Furthermore, we uncovered a potential connection between *ARID1A* and regulation of oncogenic signaling pathway and immune activity.

## 2. Materials and Methods

### 2.1. Mouse Data and Analysis

**Figure 1:**
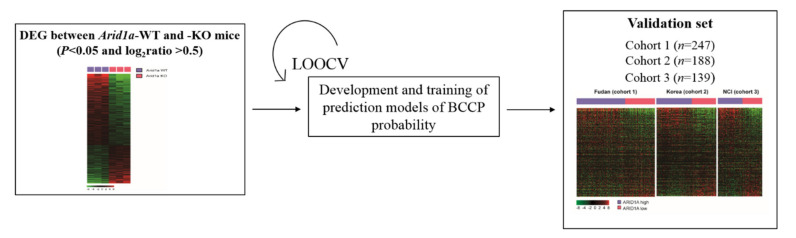
Schematic overview of derivation of *Arid1a* gene signature from mouse model and construction of Bayesian compound covariate predictor (BCCP) models. LOOCV: leave-one-out cross-validation; DEG: differentially expressed genes; WT: wild-type; KO: knockout.

Mouse gene expression data were obtained from Gene Expression Omnibus (GEO; accession number: GSE76926) with RNA-seq transcriptional profiling in liver tissues obtained after partial hepatectomy in *Arid1a*-WT and liver-specific *Arid1a*-knockout (KO) mice [15]. The mice were a mix of C57/B6 and 129. In *Arid1a* floxed mice, induced deletion between the two loxP sites produced cells lacking exon 8 of *ARID1A*, which created a frameshift mutation and induced nonsense-mediated decay in the resulting transcript. Genes whose expression differed significantly between *Arid1a*-WT and -KO mice (*p* < 0.05 and log_2_ ratio > 0.5) were considered part of the *Arid1a* gene signature, which was used for further analysis (Figure 1).

### 2.2. Gene Ontology (GO) Term Enrichment Analysis

Biological significance of differentially expressed genes (DEGs) were explored by GO term enrichment analysis including biological process, cellular component, and molecular function based on Bioconductor packages “topGO.” A *p*-value < 0.05 derived using the Kolmogorov-Smirnov test was considered to have statistical significance.

### 2.3. Immunohistochemistry on Liver Cancer Tissue

The immunohistochemistry (IHC) data was derived from existing databased, The Human Protein Atlas (https://www.proteinatlas.org) and analyzed the correlation of expression between *ARIDA1A* and alpha-fetoprotein (AFP). The AFP level is positively correlated with HCC progression and higher expression is considered to have worse prognosis.

### 2.4. Validation of ARID1A Gene Signature in Three Independent HCC Patient Cohorts

To examine the clinical relevance of low *ARID1A* expression in human HCC, the *ARID1A* gene signature was applied to gene expression data from three HCC patient cohorts: a cohort from the Fudan University Liver Cancer Institute (cohort 1; *n* = 242, GSE14520) [16,17], a Korean cohort (cohort 2; *n* = 188, GSE16757 and GSE43619), and a U.S cohort (National Cancer Institute [NCI]; cohort 3; *n* = 139, GSE1898) [18]. The baseline characteristics of the patients in the three cohorts are shown in Tables S1 and S2. Cohort 1 and 2 included Asian patients, while cohort 3 included Western HCC patients. Cohort 3 had larger tumors and more advanced stage HCC when compared to other two cohorts and shown in Table S2. 

BRB Array Tools software programs (http://linus.nci.nih.gov/BRB-ArrayTools.html) were used for gene expression data analysis and construction of a prediction model [19]. A heat map was generated using the Cluster and TreeView software programs [20], and further statistical analysis was performed using the R language (http://www.r-project.org).

Expression data from *Arid1a* WT and KO mice were used to build a classifier based on the Bayesian compound covariate predictor (BCCP) algorithm [21]. Before applying the *Arid1a* gene signature, gene expression data from the human HCC cohort were normalized and centralized as described previously [18]. The robustness of the classifier was assessed using a misclassification rate determined by leave-one-out cross-validation in the mouse set. The BCCP classifier estimated the likelihood that an individual HCC patient would have either the *ARID1A*-WT (*ARID1A*-high) or *ARID1A*-mutant (*ARID1A*-low) gene signature, and tumors were dichotomized according to Bayesian probability (cutoff of 0.5). HCC patients from all three cohorts were classified as *ARID1A*-high or *ARID1A*-low based on the BCCP predictor algorithm [19]. After categorizing patients into these two groups, patient prognoses were determined using Kaplan-Meier plots and the log-rank test.

### 2.5. TCGA Analysis Pipeline 

To extend our understanding of the molecular features of *ARID1A*-low HCC, further exploration was made through The Cancer Genome Atlas (TCGA) data portal. TCGA HCC patients were categorized as having *ARID1A*-high or *ARID1A*-low subtype HCC by the BCCP prediction method. Next, TCGA platforms were applied to these two groups to identify distinct molecular features associated with *ARID1A* mutation. The following platforms were used: (1) RNA sequencing, the Illumina HiSeq 2000 RNA Sequencing; (2) reverse-phase protein array (RPPA), the MD Anderson Cancer Center TCGA proteome characterization center RPPA core using 219 antibodies; (3) somatic mutations, the Illumina HiSeq and IlluminaGA system; (4) copy number alterations, the Affymetrix Genome-wide Human SNP Array 6.0 platform; and (5) DNA methylation profile, the Illumina Infinium HumanMethylation450 platform. 

These platforms were analyzed with the aim of gaining insight of correlation between RNA and protein data, to observe whether the copy number alteration and DNA hypermethylation could at least have partial effect on *ARID1A* expression.

### 2.6. Clinically Defined Molecular Subtypes of HCC and Associated Gene Signatures

The National Cancer Institute Proliferation (NCIP) [22], hepatic stem cell [18], silence of Hippo pathway [16], and Seoul National University Recurrence (SNUR) [23] HCC subtypes and associated gene signatures were described in earlier studies. These genes were applied to cohort 1, and co-occurrence of the subtypes with the *ARID1A*-low type was analyzed.

The six-gene interferon-gamma (IFNG6) composite score, which is associated with clinical response to pembrolizumab therapy [24] is derived from the average expression of the six genes (*CXCL9, CXCL10, IDO1, IFNG, HLA-DRA,* and *STAT1*). This six-gene signature serve as predictive biomarker in the form of a composite score in pembrolizumab treated patients where a higher score is associated with better treatment response.

The cytolytic score is based on the mean transcript levels of two key cytolytic effectors, granzyme and perforin [25] where higher cytolytic score is associated with better prognosis. The immune signature score (IS) represents 105 genes that were differentially expressed between responder and non-responder of malignant melanoma patients who were enrolled in a phase II trial of immunotherapy with MAGE-A3 antigen [26]. The signature was validated in other two cohorts of melanoma treated with anti-CTLA-4 antibody [27,28]. The number of genes used for analysis are listed in Table S3.

### 2.7. Gene Network Analysis

Next, we used gene network analysis to identify potential upstream transcription factors that regulate gene expression patterns enriched in the *ARID1A*-low subtype. The analysis used Ingenuity Pathway Analysis (IPA), and thus, was based on prior knowledge of expected effects of transcriptional factors on their target genes stored in the Ingenuity Knowledge Base. Genes whose expression differed significantly (*p* < 0.001 and log_2_ ratio > 0.5) between patients with *ARID1A*-low HCC and those with *ARID1A*-high HCC from the TCGA cohort were selected.

Gene set enrichment analysis (GSEA) was performed to identify gene sets differentially expressed in *ARID1A*-low and -high groups from the MSigDB databases (www.broadinstitue.org/msigdb).

## 3. Results

### 3.1. Development of ARID1A Gene Signature

To identify genes whose expression is highly dependent on *ARID1A* in the liver, we selected genes that had significant difference of expression between *Arid1a*-WT and *Arid1a*-KO mice. A total of 733 genes were identified (Figure S1) with 255 genes up-regulated and 478 genes down-regulated, and together these were considered the *ARID1A* gene signature for further studies Table S4. 

The biological and molecular significance of this gene signature were analyzed using GO term enrichment analysis and the results are shown in Figure S2 and GO annotation system is shown in Table S5. For biological process, the up-regulated DEGs were significantly enriched with cellular response and cytokine-mediated signaling pathway and the down-regulated DEGs are significantly enriched protein ubiquitination and mesenchymal differentiation. For molecular function, protein binding is significantly enriched in up-regulated DEGs, while molecular function regulator and enzyme molecule activity are significantly enriched in down-regulated DEGs. 

### 3.2. Clinical Significance ARID1A Gene Signature

**Figure 2:**
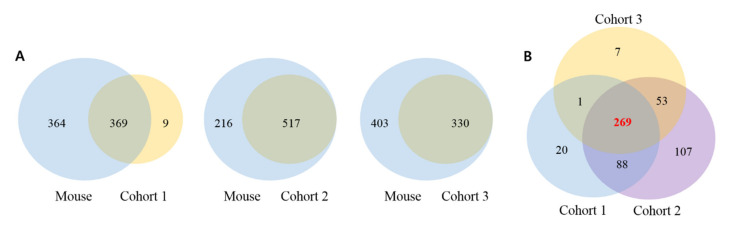
Venn diagram of mouse and human genes. (**A**) Venn diagram of mouse and human genes that are commonly expressed and (**B**) Venn diagram human genes that are shared among three cohorts.

To examine the clinical relevance of low *ARID1A* expression in HCC, the *ARID1A* gene signature was applied to three independent HCC cohorts. The number of genes in common between each cohort as well as gene signature with all cohorts are shown in Figure 2A,B. 

The heatmaps of genes that were shared between the mouse gene signature and the human gene signature are shown in Figure 2A. Of the 733 genes identified by this analysis, 269 genes were shared by all three cohorts (Figure 2B). The 269 genes that are commonly share by three cohorts were applied for GO term enrichment analysis and the results are shown in Figure S3, while the GO annotation system is shown in Table S6.

For biological process, the up-regulated DEGs were significantly enriched with cellular protein modification system, cell death, and intracellular and cell junction organization, while down-regulated genes were enriched with positive regulation of immune system process, regulation of mitotic cell cycle, and cellular macromolecule catabolic process. For molecular function, protein binding is significantly enriched in up-regulated DEGs, while molecular function regulator and enzyme regulator activities are significantly enriched in down-regulated DEGs. 

In cohort 1, 94 of 242 patients (38.8%) were categorized to the *ARID1A*-low group; in cohort 2, 63 of 139 patients (45.3%) were placed in the *ARID1A*-low group; in cohort 3, 76 of the 188 patients (40.4%) were placed in the *ARID1A*-low group. After dichotomizing patients in these three cohorts into *ARID1A*-high and -low groups, generally, patients who were placed in the *ARID1A*-low group had higher baseline alpha-fetoprotein (AFP) levels (AFP > 300 ng/mL; cohort 1, 60% vs. 37%, *p* = 0.001; cohort 2, 45% vs. 19%, *p* < 0.001) and advanced HCC stage (stage III; cohort 1, 33% vs. 14%, *p* = 0.011; cohort 2, 34% vs. 26%, *p* = 0.002; Table S1). Consistent with the clinical findings, the IHC staining of five HCC tumors revealed four tumors with low expression of *ARID1A* had high expression of AFP (Figure S4).

**Figure 3:**
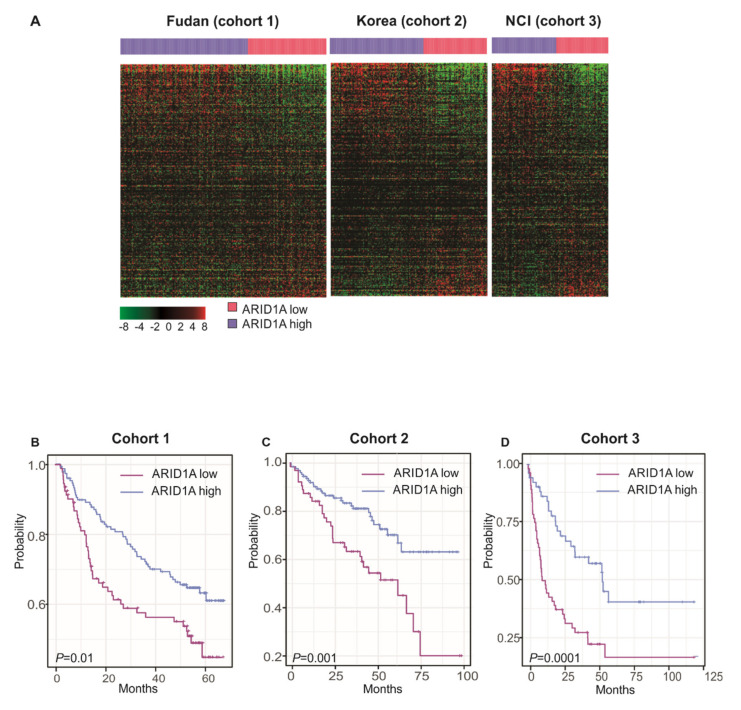
*ARID1A* gene expression signature and overall survival analysis in patients with hepatocellular carcinoma stratified by *ARID1A* activity. (**A**) Heatmaps of the 269 genes (of the 733 genes of the *ARID1A* gene signature) that were shared among three different HCC cohorts. The *ARID1A* signature could effectively discriminate the survival rates of *ARID1A*-low and *ARID1A*-high HCC subtypes, with lower survival rates in the mutated *ARID1A*-low group than in wild-type *ARID1A*-high group for (**B**), cohort 1 (*n* = 242); (**C**), cohort 2 (*n* = 188); and (**D**), cohort 3 (*n* = 139).

We next validated the prognostic association of the *ARID1A* gene signature in the three independent cohorts. This analysis identified a significant difference in survival between the patients in the *ARID1A*-high group and those in the *ARID1A*-low group. Overall, patients in the *ARID1A*-low group had a lower survival rate than those in the *ARID1A*-high group (Figure 3B–D). Furthermore, *ARID1A*-low subtype remained as significant predictor when AFP level and tumor size were included in multivariate analysis (HR 1.413, CI 1.234-1.619, *p* < 0.01).

### 3.3. Association of ARID1A Gene Signature with Other Gene Signatures

First, *ARID1A*-low and *ARDI1A*-high groups of cohort 1 were compared with NCIP subtypes (A or B). The NCIP gene signature was derived by using unsupervised analysis of genome-level expression data from human HCC tissues where subtype A was associated with poor prognosis. When this signature was applied, 67 of 94 (71.3%) *ARID1A*-low group patients and 21 of 153 (13.7%) of *ARID1A*-high group patients were categorized to the poor-prognosis NCIP A subtype (*p* < 0.001, chi-square test).

**Figure 4:**
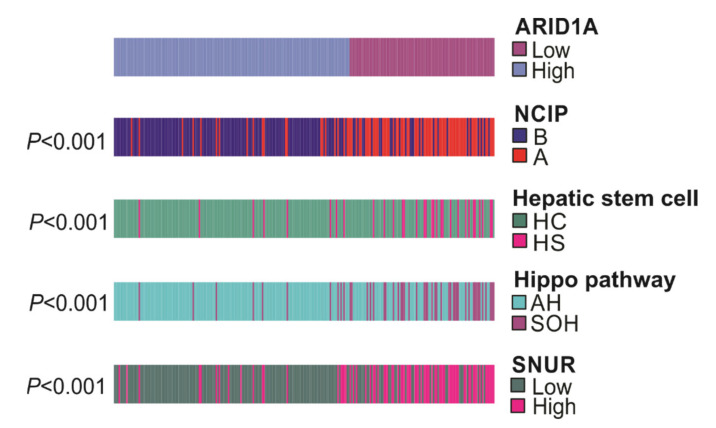
Association of *ARID1A* activity with previously identified molecular subtypes of hepatocellular carcinoma.

In addition to the NCIP gene signature, other signatures applied were (1) the hepatic stem cell gene signature (vs. hepatocytes), derived from expression patterns resembling fetal hepatic stem cells, which was associated with poor prognosis; (2) the silence of Hippo gene signature (vs. activated Hippo), where the HIPPO pathway is a tumor suppressor in the liver and its inactivation is known to be associated with poor prognosis; and (3) the SNUR recurrence-high gene signature (vs. recurrence-low), which was derived using supervised approaches in selecting genes associated with early disease recurrence after curative-intent treatment. When each of these signatures was applied, the *ARID1A*-low HCC subtype was consistently associated with poor-prognosis and high-recurrence subtypes (Figure 4). Furthermore, *ARID1A* gene signature remained significant in multivariate analysis when compared with previously reported gene signatures (Table S7).

The *ARID1A*-low HCC subtype was significantly associated with the NCI high-proliferation subtype (NCIP A), the hepatic stem cell (HS) subtype, the silence of Hippo pathway (SOH) subtype, and the high-recurrence (SNUR high) subtype (all, *p* < 0.001).

### 3.4. ARID1A-Low Subtype Cancer in Multi-Platform Analysis

Because the survival analysis and comparison with previously published gene signatures indicated that the *ARID1A*-low subtype is significantly correlated with poor prognosis, we next investigated the molecular characteristics of HCCs by *ARID1A* subtype using the multi-platform data accessed through the TCGA data portal. When the *ARID1A* gene signature was applied to 371 patients in the TCGA cohort, 170 patients (46%) were classified to the *ARID1A*-high group and 201 patients (54%) to the *ARID1A*-low group. The pattern of survival in the TCGA cohort was similar to that in the three validation cohorts, with a lower survival rate and higher recurrence rate in the *ARID1A*-low group (log rank, *p* = 0.0017 and *p* = 0.004 respective; Figure S5A,B).

**Figure 5:**
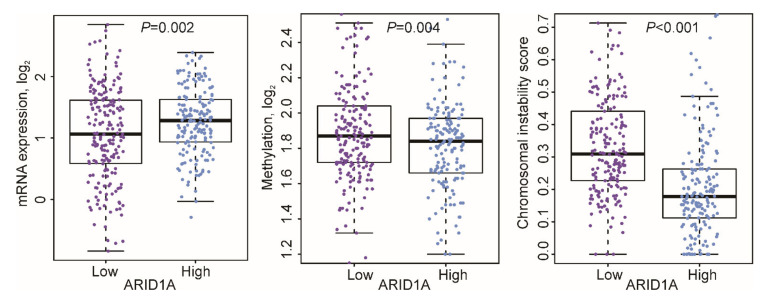
Distribution of mRNA expression, methylation levels, and chromosomal instability of the *ARID1A* gene in the *ARID1A*-low and *ARID1A*-high groups. The *ARID1A*-low subtype was associated with significantly lower *ARID1A* mRNA expression and greater methylation level and chromosomal instability.

**mRNA, protein, and methylation.** Analysis of mRNA expression data revealed that the *ARID1A* mRNA expression level was significantly lower in the *ARID1A*-low group than in the *ARID1A*-high group (mean log_2_ 1.05 vs. 1.27, *p* = 0.002). The methylation level and chromosomal instability were significantly greater in the *ARID1A*-low group than in the *ARID1A*-high group (*p* = 0.004 and *p* < 0.001; Figure 5).

Since protein expression level would more accurately reflect the characteristics of *ARID1A*-low HCC, we analyzed the proteins that were associated with the *ARID1A*-low group. Of the 371 patients in the TCGA group, 181 had available RPPA data, and these data were used for the protein analysis. *ARID1A* protein level was positively correlated with mRNA expression levels (Pearson coefficient 0.36, *p* < 0.001) (Figure S6). Further analysis was performed to observe epigenetic modifications that decreased *ARID1A* mRNA expression. Increased copy number alteration was associated with increased mRNA expression levels (Pearson coefficient 0.32, *p* < 0.001), while hypermethylation was negatively correlated with mRNA expression levels (Pearson coefficient -0.22, *p* < 0.001) (Figure S6). 

**Figure 6:**
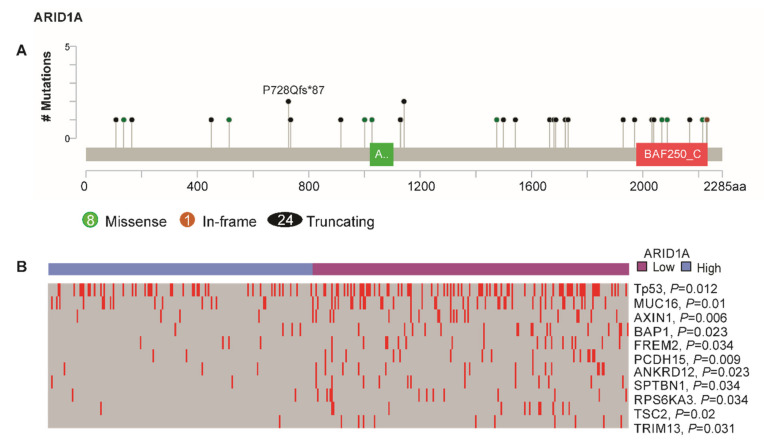
Somatic mutations in *ARID1A* and distribution of frequently mutated tumor suppressor genes in *ARID1A*-low and *ARID1A*-high groups. (**A**) Of 367 patients with somatic mutation data, 33 patients (9%) had an *ARID1A* mutation. A total of 24 truncating mutations, eight missense mutations, and one in-frame deletion were observed (www.cbioportal.org/). (**B**) Frequently-mutated tumor suppressor genes in HCC such as *TP53*, *BAP1*, *AXIN1,* and *TSC2* were observed more frequently in the *ARID1A*-low subtype than in the *ARID1A*-high subtype (all, *p* < 0.05).

**Somatic mutations.** To consolidate our findings indicating that our *ARID1A* gene signature truly identifies *ARID1A*-low subtypes with greater *ARID1A* mutation, further analysis was done for somatic mutations. Of the 371 patients, somatic mutation data was available for 367; 33 of those (9%) had *ARID1A* somatic mutation. The presence of *ARID1A* mutation in *AIRD1A*-low subtype was 71%, while it was 29% in *ARID1A*-high subtype (*p* = 0.054). Truncation was the most common type (72.7%) of mutation, followed by missense (24.3%) and in-frame (3%) mutations (Figure 6A). Furthermore, mutations of *TP53*, *AXIN1,* and *TSC2* were significantly more prominent in the *ARID1A*-low group (all, *p* < 0.05; Figure 6B).

### 3.5. Correlation of ARID1A Gene Signature with Immune Signature

**Figure 7:**
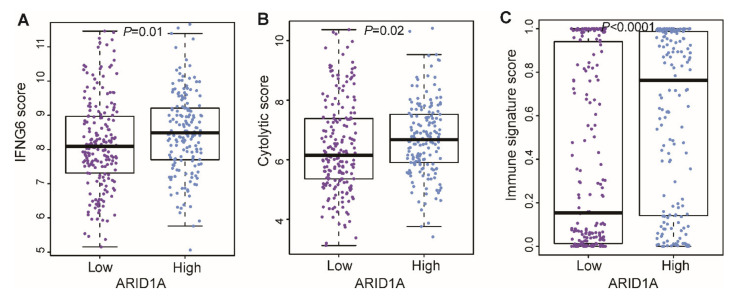
Distribution of immune activity scores in the *ARID1A*-low and *ARID1A*-high groups. The immune scores were derived from (**A**) the six-gene interferon-gamma (IFNG6) composite score; (**B**) the cytolytic score; and (**C**) the immune signature score. All were significantly lower in the *ARID1A*-low group.

Since patients with the *ARID1A*-low subtype of HCC had a poorer prognosis, we next assessed the immune status of the patients in the two *ARID1A* expression groups. The IFNG6 composite score, which reflects overall immune activity and predicts response to anti-PD-1 (pembrolizumanb) treatment in cancer patients, was calculated on the basis of the genes reported by Seiwert et al. [24] IFNG6 score was significantly lower in the *ARID1A*-low group than in the *ARID1A*-high group (*p* = 0.01; Figure 7A). Similar results were observed for the cytolytic activity score [25] and the IS score [26], where lower scores were significantly associated with the low-*ARID1A* subtype (*p* = 0.02 and *p* < 0.001, respectively; Figure 7B,C).

As immune checkpoint genes control the balance of stimulatory and inhibitory signals and play critical roles in regulating T-cell activities, we further compared the expression of ligands and receptors for stimulatory and inhibitory signals between *ARID1A*-low and *ARID1A*-high subtypes. Of the nine immune-stimulatory genes studied, four (CD28, CD40, CD226, and IL2RB) were significantly suppressed in the *ARID1A*-low group, while inhibitory genes PD-L1 (CD276) and LAG3 were highly expressed in the *ARID1A*-high group (all, *p* < 0.01; Figure S7). 

### 3.6. Functional Pathway Analysis

To gain better insight into the molecular characteristics of the *ARID1A* signature, genes that differed between the *ARID1A*-high and -low groups (*p* < 0.001 and log_2_ ratio < 0.5), a total of 2503 genes, were selected for functional study based on the Ingenuity Knowledge Base repository [29]. Direct upstream transcription regulators of the *ARID1A* signature were genes associated with cell cycle, including *SMAD7*, *CCND1, MYC,* and the E2F group comprising *E2F1* (with activated *z*-scores > 2), while *TP53, SMARCB1, SMARCA4*, *CDKN2A, SMAD3,* and *CTNNB1* were significantly inhibited (*z*-scores < -2) (Table S7).

## 4. Discussion

The SWI/SNF complex represents a novel link between chromatin remodeling and tumor suppression. Recurrent mutations in subunits of the complex have been identified in various cancers [30]. *ARID1A*, a subunit of SWI/SNF complexes that controls how much “read access” the cellular transcription machinery has to DNA sequences, can have profound consequences on gene expression, and genes encoding chromatin-remodeling proteins are some of the most frequently mutated genes in human cancer [31]. However, few studies of the association between *ARID1A* and hepatocarcinogenesis have been published, and a recent study reported controversial results [12,13,14]. Therefore, our purpose was to identify the role of *ARID1A* as a predictor of outcome in HCC patients with the intent of identifying a mechanism underlying the suppression of *ARID1A* using a bioinformatic approach. Since *ARID1A*’s biological activity is best reflected in gene expression level, we have derived signature from *Arid1a* KO mouse dataset and applied to human dataset to stratify HCC tumors according to *ARID1A* activity. Since the best-known molecular activity of *Arid1a* is the regulation of gene expression through chromatin remodeling, the transcriptomic signature of *Arid1a* KO mice would best reflect its biological activity. Functional conservation of *Arid1a*’s role in liver as tumor suppressor is well-documented in a recent study from Zhu et al., demonstrating that *Arid1a* is one of frequently mutated genes in chronically diseased liver tissues and loss of Arid1a promote clonal expansion in damaged mouse liver [12]. Furthermore, its tumor suppressor activity has also been demonstrated in mouse models.

Based on the analysis, first we confirmed that an *ARID1A*-related gene signature is effective in identifying HCC patients with poor prognosis. Patients in *ARID1A*-low subtype HCC had a significantly lower survival rate than *ARID1A*-high subtype in all four cohorts studied. Furthermore, when HCC subtypes derived from our *ARID1A* gene signature were systematically compared with previously validated gene signatures, we confirmed the correlation of our *ARID1A*-low subtype with the poor-prognosis subtypes NCIP A, which reflected high proliferation; hepatic stem cell, where the stem cell subtype is known to be associated with poor prognosis in all cancers; silence of the Hippo pathway, where the Hippo pathway is indispensable in restricting cell growth and proliferation and its inactivation leads to poor prognosis; and recurrence-high SNUR. 

The exact molecular mechanism underlying low *ARID1A* mRNA and protein expression is still not clear. Although the correlation between mRNA and protein level was not prominent with coefficient of 0.36, this result is in line with previous study by Chen et al., where positive correlation with coefficient averaging ρ = 0.3 was observed, while a median value of 0.45 was reported in another study [32,33]. In the present study, the mechanism of tumor suppression by *ARID1A* was investigated using high-throughput platform data obtained from the TCGA portal. From the results, we speculated that the loss of *ARID1A* was due to hypermethylation, which is in accordance with a previous study proposing that promoter hypermethylation of the *ARID1A* gene is responsible for the low *ARID1A* mRNA expression in invasive breast cancers [34]. Another reason for the low *ARID1A* expression could be increased copy number alteration, leading to poor prognosis of HCC. Published evidence that gains in chromosomes 1q and 8q are linked to hepatocarcinogenesis [10,35] is supported by our observations in *ARID1A*-low subtype. The mutation rate (9%) of ARID1A in our present study is similar to study reported by Zucmann-Rossi et al., which showed 4–17% of *ARID1A* mutation and 10% according to study by Fujimoto et al. [36,37].

Recent success for targeted therapies against immune checkpoints such as CTLA-4, PD-1 (PDCD1), and PD-L1 (CD274) [38,39] led us to characterize the immune microenvironment in HCC according to *ARID1A*-low and -high subtypes. One of these immune checkpoint–targeted agents, pembrolizumab, which targets PD-1, a negative co-stimulatory receptor and a strong inhibitor of T-cell response, is under clinical trial for treatment of HCC [40]. When we assessed immune activity indicative of a response to pembrolizumab by the IFNG6 score, the *ARID1A*-low subtype had a lower IFNG6 score than the *ARID1A*-high subtype. Consistent with the lower IFNG6 score in the *ARID1A*-low subtype was the lower immune cytolytic activity, based on transcript levels of two key cytolytic effectors, granzyme A and perforin, which are dramatically up-regulated upon CD8+ T cell activation [41] during productive clinical responses to anti-CTLA-4 and anti-PD-L1 immunotherapies [14,42], were also lower in the *ARID1A*-low group. Likewise, IS score, derived from 105 genes based on response to anti-CTLA-4 treatment in melanoma patients, was lower in the *ARID1A*-low group. Low *ARID1A* expression had suppressed expression of immune-stimulatory genes, while it increased expression of inhibitory genes. Since immune-stimulatory genes are known to predict better prognosis and are found to be protective factor in cancer, while immune inhibitory genes lead to tumor promoting signals and have negative correlation with survival, our results are in accordance with the already known findings.

Gene network analysis revealed that cell proliferation and cell cycle dysregulation were activated as a consequence of *ARID1A* loss. Since *ARID1A* is a subunit of the SWI/SNF complex that coordinates activity of the proteins of the complex, it is responsible for altering the chromatin structure required to facilitate several cellular functions, including transcription, DNA synthesis, and DNA damage repair [43]. Therefore, it is not surprising that tumor suppressor genes such as *TP53* and cyclin-dependent kinase genes such as *CDKN2A* are inactivated in the *ARID1A*-low subtype. *TP53* down-regulation was observed in every analysis we did, including RPPA, somatic mutation analysis, and copy number alterations. The tumor suppressor function of *ARID1A* through collaboration with *TP53* is disrupted by loss of *ARID1A* and this change could have led to disruption in *TP53*-regulated genes such as *CDKN1A* and *SMAD3*, which were all inhibited in IPA analysis. All these alterations in cell cycle–regulatory genes could have eventually caused hepatocarcinogenesis with poor prognosis.

Sun et al. reported that *ARID1A* promotes development of HCC at a very early stage while preventing HCC progression to metastatic tumor [12]. Our observation with established HCC agrees well with their results, supporting the idea that *ARID1A* has tumor suppressor activity in the later stage of HCC. Another recent study, by Hu et al. [14] using mouse and human data, revealed that loss of *ARID1A* was associated with increased angiogenesis and poor prognosis after HCC had established. *ARID1A* was expressed in the nucleus of all hepatocytes in normal liver and precancerous lesions, but *ARID1A* expression was heterogeneous in HCC. In accordance with their findings, our study showed an increased probability score for *ARID1A* loss in advanced stage HCC compared to early stage HCC, indicating that *ARID1A* deletion is likely to occur in late-stage disease. 

Our present study was based on an in silico analysis and has a limitation in the functional validation of our results. Despite this limitation, our present findings set the stage to explore the potential of *ARID1A* in translational application in the era where molecular profiling of HCC from the TCGA data is almost complete, while its translation into the clinic remains beyond the horizon. Future studies are required to establish the molecular relationships between *ARID1A* suppression and the identified pathway. 

## 5. Conclusions

The low immune activity along with activation of genes that are associated with HCC development in low-*ARID1A* subtype indicate that *ARID1A* may have tumor suppressive activity and suggests the possibility of *ARID1A* as a prognostic biomarker in HCC patients.

## Supplementary Materials

The following are available online at https://www.mdpi.com/2073-4409/9/9/2002/s1, Figure S1: Heatmap of mouse *Arid1a* gene signature. Figure S2: GO enrichment analysis result of up-regulated and down-regulated DEGs of *Arid1a* KO mouse. BP, biological process; CC, cellular component; MF, molecular function. Figure S3: GO enrichment analysis result of up-regulated and down-regulated DEGs of human ARID1A gene signature. BP, biological process; CC, cellular component; MF, molecular function. Figure S4: Negative correlation of *ARID1A* and AFP expression in HCC tissues. Figure S5: Kaplan Meier plot for overall and recurrence free survival analysis. Figure S6: Correlation between ARID1A mRNA expression level, copy number alteration, and methylation. Figure S7: Expression level of immune stimulatory and inhibitory genes. Table S1: Clinical characteristics of patients in the validation cohorts according to high and low ARID1A. Table S2: Tumor factors according to three independent cohorts. Table S3: Prognostic molecular subtypes of HCC and associated gene expression signatures. Table S4: Arid1a gene signature that is derived from 733 genes that differed with *p* < 0.05 and log2 ratio > 0.5 between Arid1a WT and KO mice. Table S5: GO annotation of mouse *Arid1a* gene signature. Table S6: GO functional annotation of human *ARID1A* gene signature. Table S7: Prognostic gene signature.
